# Lead Pipe

**Published:** 1879-11

**Authors:** 


In moving into a new house, supplied with lead pipes, no water through them should be used for at least one month, nor after a house has been unused for a week or more; the cook should be required to let the water run with its full force, until she can count thirty, before the water is taken for cooking or drinking purposes. All lead pipes lined, it matters not with what material, are dangerous and deadly, for they will almost certainly crack, and any two metals communicating with water, however pure, will corrode.

